# Using RNA-Sequencing Data to Examine Tissue-Specific Garlic Microbiomes

**DOI:** 10.3390/ijms22136791

**Published:** 2021-06-24

**Authors:** Yeonhwa Jo, Chang-Gi Back, Kook-Hyung Kim, Hyosub Chu, Jeong Hun Lee, Sang Hyun Moh, Won Kyong Cho

**Affiliations:** 1 Research Institute of Agriculture and Life Sciences, College of Agriculture and Life Sciences, Seoul National University, Seoul 08826, Korea; yeonhwajo@gmail.com (Y.J.); kookkim@snu.ac.kr (K.-H.K.); 2 Horticultural and Herbal Crop Environment Division, National Institute of Horticultural and Herbal Science, RDA, Wanju 55365, Korea; plantdoctor7@korea.kr; 3 R&D Division, BERTIS Inc., Seongnam-si 13605, Korea; hyosubchu@gmail.com; 4 Plant Cell Research Institute of BIO-FD&C Co., Ltd., Incheon 21990, Korea; jhlee@biofdnc.com

**Keywords:** pepper, fruit, microbiome, bacteria, fungi, viruses, metagenomics, metatranscriptomics

## Abstract

Garlic (*Allium sativum*) is a perennial bulbous plant. Due to its clonal propagation, various diseases threaten the yield and quality of garlic. In this study, we conducted in silico analysis to identify microorganisms, bacteria, fungi, and viruses in six different tissues using garlic RNA-sequencing data. The number of identified microbial species was the highest in inflorescences, followed by flowers and bulb cloves. With the Kraken2 tool, 57% of identified microbial reads were assigned to bacteria and 41% were assigned to viruses. Fungi only made up 1% of microbial reads. At the species level, *Streptomyces lividans* was the most dominant bacteria while *Fusarium pseudograminearum* was the most abundant fungi. Several allexiviruses were identified. Of them, the most abundant virus was garlic virus C followed by shallot virus X. We obtained a total of 14 viral genome sequences for four allexiviruses. As we expected, the microbial community varied depending on the tissue types, although there was a dominant microorganism in each tissue. In addition, we found that Kraken2 was a very powerful and efficient tool for the bacteria using RNA-sequencing data with some limitations for virome study.

## 1. Introduction

Garlic (*Allium sativum*) is a perennial bulbous plant that is famous for its flavorful bulbs. Garlic is a member of the genus *Allium* and is closely related to the chive, leek, shallot, onion, rakkyo, and scallion. Garlic cloves have an intense flavor and aroma and are frequently used for various cuisines and medicines around the world. In addition, garlic flower stalks, known as scapes, are also consumed as a vegetable. Garlic is usually propagated by planting individual cloves taken from entire garlic bulbs during the fall season (from October to November). Although garlic is regarded as very easy to grow, various diseases threaten the yield and quality of garlic. Bacteria such as *Pantoea*, *Pseudomonas*, *Burkholderia*, and *Xanthomonas* species cause flower stalk and leaf necrosis, leaf spot, slippery skin, and leaf blot symptoms in the infected garlic [1,2,3]. The well-known fungal diseases for garlic are white rot by *Sclerotium cepivorum*, vegetable rot by *Penicillium*, *Mucor* or *Rhizopus* species, fusarium bulb and basal rot by *Fusarium* species, botrytis neck ort caused by *Botrytis porri*, embellisia skin blotch by *Embellisia allii*, and rust by *Puccinia allii* [4,5,6,7,8,9]. Due to its clonal propagation, garlic is co-infected by diverse viruses causing mosaic, yellow spot, and dwarf symptoms [10,11]. The most common viruses infecting garlic are allexiviruses such as garlic virus A (GarVA), garlic virus B (GarVB), garlic virus C (GarVC), garlic virus D (GarVD), garlic virus E (GarVE), garlic virus X (GarVX), and shallot virus X (ShVX). In addition, three potyviruses—onion yellow dwarf virus (OYDV), leek yellow stripe virus (LYSV), and shallot yellow stripe virus (SYSV)—and two carlaviruses—garlic common latent virus (GCLV) and shallot latent virus (SLV)—are commonly identified in garlic.

In general, to diagnose plant diseases, plant samples showing disease symptoms are collected and cultivated on the specific medium for bacteria or fungi. After that, the nucleic acids are extracted from the cultivated bacterial colonies or fungal mycelia. Using primers for the bacterial 16S ribosomal RNA (rRNA) gene or fungal internal transcribed spacer (ITS) region of rRNA, polymerase chain reaction (PCR) is carried out followed by the sequencing of amplified PCR fragments [12]. The cultivation-dependent method has been usefully applied for the diagnosis of major plant pathogens in a given plant sample [13]. Recently, next-generation sequencing techniques have been able to facilitate the identification of both cultural and uncultivable pathogens and reveal the microbiome community in a given plant sample [14].

DNA shotgun sequencing and PCR-amplified marker genes have been widely used for the microbiome study of bacteria and fungi. By contrast, viruses do not have common marker genes, which is the main obstacle for virome study [15]. Recently, an RNA-sequencing based approach with various library types such as total RNA library after deleting ribosome, small RNA library, double-stranded RNA library, and mRNA library has been used as a powerful tool to study plant RNA viromes [16]. It is generally difficult to enrich viral particles from the collected samples, so most sequence reads by RNA-sequencing should be derived from the plant hosts. The proportion of viral reads by RNA-sequencing might be diverse depending on the library type. In addition, the transcriptome by RNA-sequencing very often contains microbial sequences derived from bacteria and fungi.

In this study, we conducted in silico analysis to identify microorganisms, bacteria, fungi, and viruses using garlic RNA-sequencing data from six different tissues.

## 2. Results

### 2.1. Removal of Poor-Quality Reads and the Reads from the Garlic

We retrieved SRA data from a previous study that conducted garlic transcriptome study possessing six different garlic tissues [17]. By quality trimming, the proportion of removed reads ranged from 4.72% (flowers) to 9.96% (leaves) (Table 1). Next, we deleted sequences derived from garlic including nuclear, chloroplast, and mitochondrial genomes. The proportion of the sequence reads derived from the garlic ranged from 53.02% (leaves) to 74.34% (flowers). After removing low-quality reads and garlic-associated reads, the proportion of filtered reads ranged from 25.65% (flowers) to 46.98% (leaves). Clean reads were subjects for the identification of microorganisms.

### 2.2. Identification of Microorganisms by Kraken2

**Figure 1:**
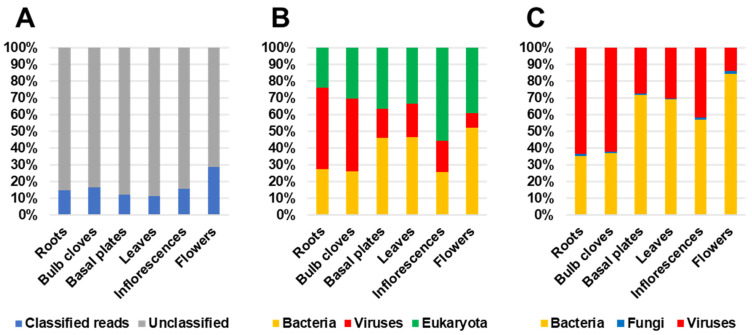
Proportion of sequenced reads in six different garlic tissues. (**A**) Proportion of classified and unclassified reads. (**B**) Proportion of bacteria-, virus-, and eukaryota-associated reads. (**C**) Proportion of bacteria-, fungi-, and virus-associated reads in six different garlic tissues.

**Figure 2:**
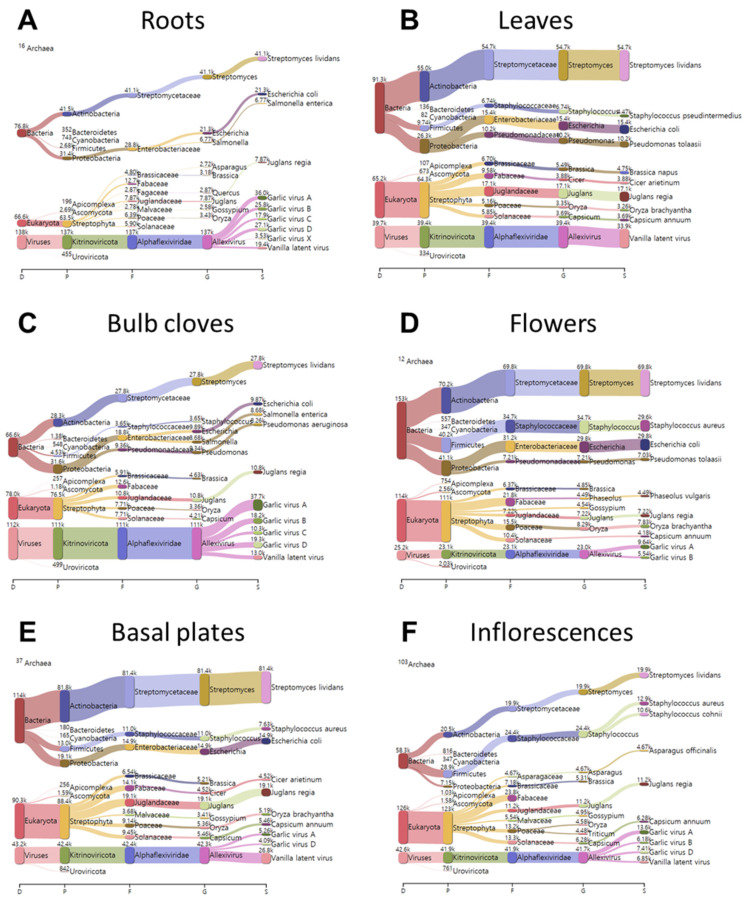
Taxonomy classification in six different garlic tissues using Kraken2. Clean reads were used for taxonomy classification using Kraken2. Sankey diagrams visualize identified taxonomy at four different levels: phylum, family, genus, and species, along with the number of assigned reads in six different samples (**A**) roots, (**B**) leaves, (**C**) bulb cloves, (**D**) flowers, (**E**) basal plates, and (**F**) inflorescences.

We identified three different groups of plant microorganisms, bacteria, fungi, and viruses using the Kraken2 program [18]. Unexpectedly, the majority of clean reads were unknown sequences (unclassified) (Figure 1A). The proportion of classified reads was very low, ranging from 11.56% (leaves) to 28.99% (flowers). The classified reads were mostly assigned to bacteria, viruses, and eukaryota (Figure 1B). Most of the reads assigned to eukaryota were derived from plants, although fungal reads were also identified (Figure 1B). The Kraken2 database did not possess the garlic genome data. Thus, plant-associated reads were assigned to diverse plant species such as *Asparagus officinalis*, *Brassica napus*, *Cicer arietinum*, *Juglans regia*, *Oryza brachyantha*, *Phaseolus vulgaris*, and *Capsicum annuum* (Figure 2).

In roots (48.9%) and bulb cloves (43.6%), the proportion of virus-associated reads was high among six different tissues (Figure 1C). The proportion of bacterial reads was high in basal plates (46.1%) and leaves (46.5%), while the proportion of eukaryota-associated reads was high in inflorescences (55.4%) and flowers (39%). Next, we examined the proportion of three different microorganisms including bacteria, fungi, and viruses in six different tissues (Figure 1C). The proportion of viral reads was very high in roots (63.3%) and bulb cloves (62.1%) but was low in flowers (13.9%). The proportion of bacterial reads was the highest in flowers (84.7%), followed by basal plates (71.9%) and leaves (69.3%). The proportion of fungal reads was relatively very low, ranging from 1.58% (inflorescences) to 0.55% (leaves).

### 2.3. Rarefaction Analysis

**Figure 3:**
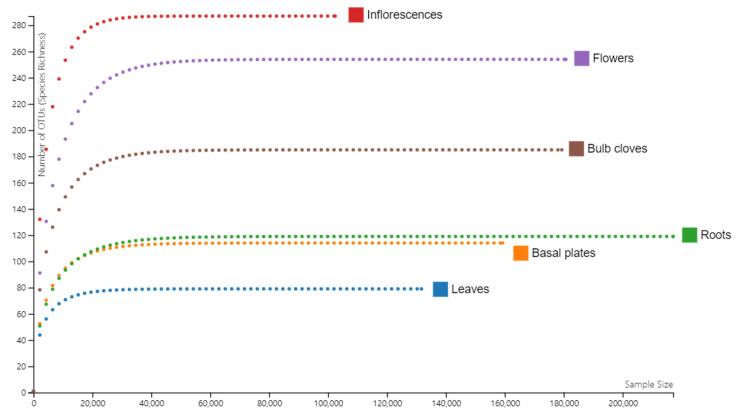
Rarefaction curves of microbiomes in six different garlic tissues.

We examined species richness of identified bacteria, fungi, and viruses in six different tissues (Figure 3). The number of identified species was the highest in inflorescences, followed by flowers and bulb cloves. We identified a very low number of microbial species from the leaves. The species richness for roots and basal plates was comparable between two tissues.

### 2.4. Alpha Diversity of Identified Microorganisms

**Figure 4:**
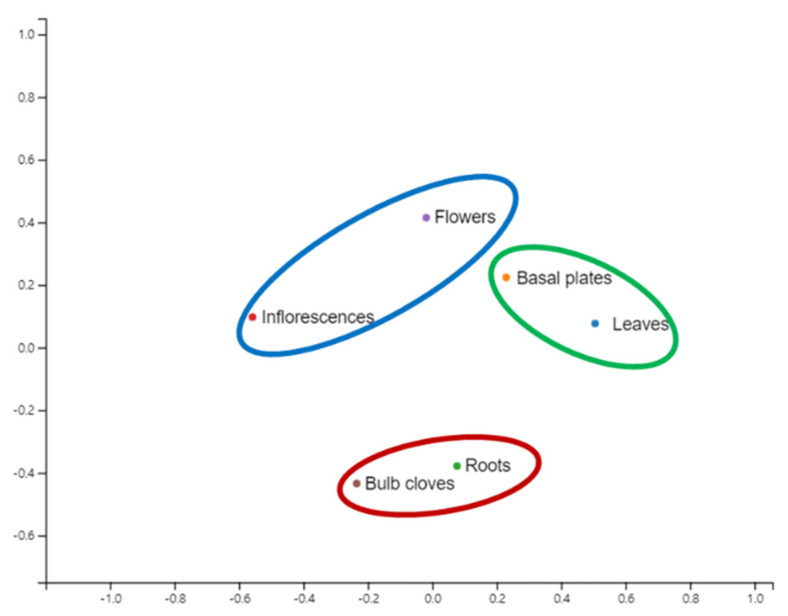
Non-metric multidimensional scaling (NMDS) of identified microorganisms, bacteria, fungi, and viruses at the species level. NMDS was conducted utilizing the scikit-learn package with Bray–Curtis distance metric.

We examined the alpha diversity of identified microorganisms based on species in six different tissues (Table 2). Among the six different tissues, inflorescences showed the highest diversity by all three indexes. By the Shannon index, the alpha diversity was low in basal plates (1.85) and leaves (1.81), whereas it was high in inflorescences (2.95) and bulb cloves (2.67). Similarly, the alpha diversity by the Simpson and inverse Simpson indexes was lowest in basal plates and highest in inflorescences. Based on microbial species, we visualized the similarity among six different libraries by non-metric multidimensional scaling (NMDS) method (Figure 4). Roots and bulb cloves were closely related, and basal plates and leaves were grouped together.

### 2.5. Major Microorganisms Identified by Kraken2

**Figure 5:**
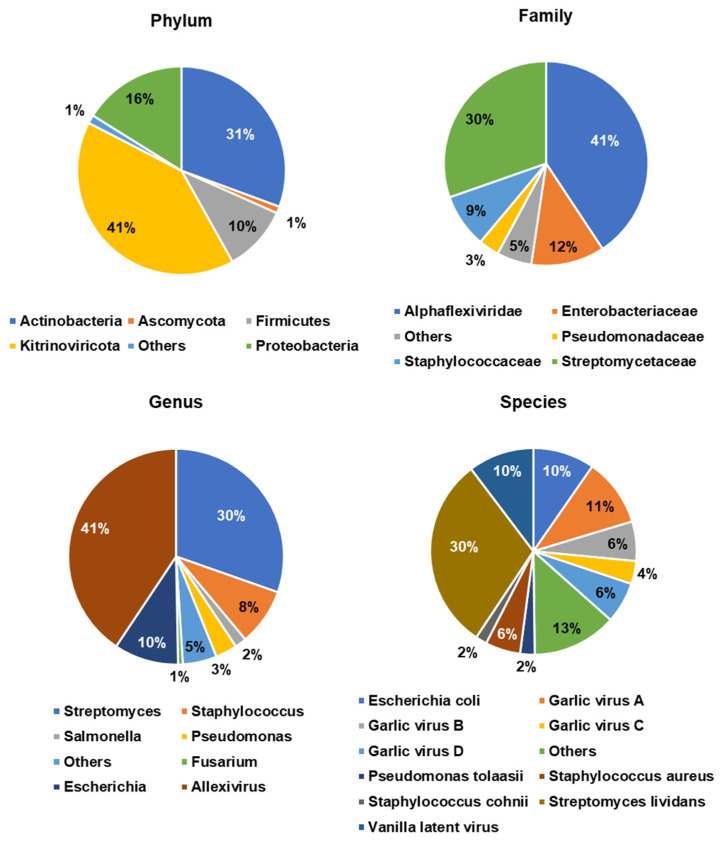
Major identified microorganisms at different taxonomy levels including phylum, family, genus, and species identified by Kraken2.

We examined the proportion of identified microorganisms according to four different taxonomy levels (Figure 5). Interestingly, the major phylum was Kitrinoviricota (41%), followed by Actinobacteria (31%), Proteobacteria (16%), Firmicutes (10%), and Ascomycota (1%). At the family level, 41% of classified reads belonged to the family Alphaflexiviridae and 30% of classified reads were identified as Streptomycetaceae. We also identified Enterobacteriaceae (12%), Staphylococcaceae (9%), and Pseudomonadaceae (3%). At the genus level, Allexivirus (41%) was a major genus, followed by Streptomyces (30%), Escherichia (10%), and Staphylococcus (8%). At the species level, 30% of classified reads were identified as Streptomyces lividans. We also identified several viral species such as GarVA (11%), vanilla latent virus (10%), garlic virus B (6%), garlic virus D (6%), and garlic virus C (4%).

### 2.6. Comparison of Identified Bacteria in Different Garlic Tissues

**Figure 6:**
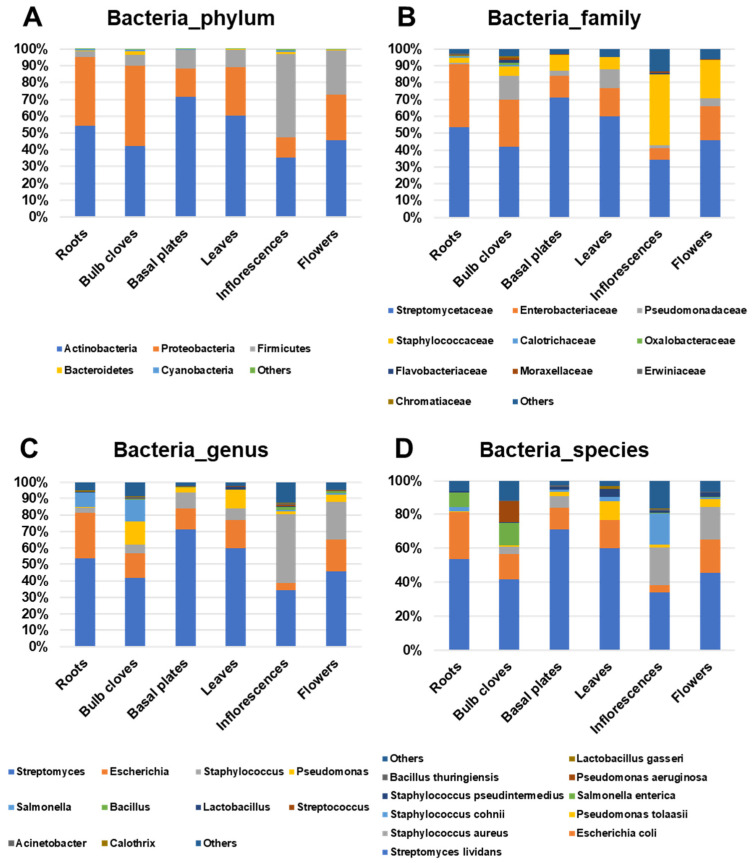
Proportion of identified bacterial reads in each tissue at four different taxonomy levels: (**A**) phylum, (**B**) family, (**C**) genus, and (**D**) species.

We compared the proportion of identified bacteria in six different garlic tissues (Figure 6). In roots and bulb cloves, *Actinobacteria* and *Proteobacteria* were identified as two major bacterial phyla (Figure 6A). *Actinobacteria* was dominant in basal plates (71.5%) and leaves (60.2%). *Firmicutes* was the third most dominant bacterial phylum in all tissues except inflorescences. In inflorescences, the *Firmicutes* (49.6%) was identified as the dominant bacterial phylum followed by *Actinobacteria* (35.2%) and *Proteobacteria* (12.3%). In flowers, *Actinobacteria* (45.9%) was the major phylum followed by *Proteobacteria* (26.8%) and *Firmicutes* (26.2%).

At the family level, *Streptomycetaceae*, *Enterobacteriaceae*, *Pseudomonadaceae*, and *Staphylococcaceae* were identified as four major bacteria families (Figure 6B). Of them, *Streptomycetaceae* was the major bacterial family in all tissues, ranging from 41.8% (bulb cloves) to 71.1% (basal plates), except inflorescences (34.3%). Bacteria belonging to the family *Enterobacteriaceae* were frequently identified in roots (37.5%), bulb cloves (28.2%), and flowers (20.4%). Bacteria in the family *Pseudomonadaceae* were abundantly present in bulb cloves (14.1%) and leaves (11.2%). Bacteria in the family *Staphylococcaceae* were highly identified in inflorescences (42.0%) and flowers (22.7%).

At the genus level, *Streptomyces*, *Escherichia*, *Staphylococcus*, *Pseudomonas*, and *Salmonella* were identified as major bacterial genera (Figure 6C). Of them, *Streptomyces* was the most dominant bacteria in most tissues, ranging from 34.3% (inflorescences) to 71.1% (basal plates). *Escherichia* was the second most dominant bacterial genera, ranging from 4.3% (inflorescences) to 27.8% (roots). *Staphylococcus* were identified in most tissues; however, the proportion of *Staphylococcus* was very high in inflorescences (42%). Bacteria belonging to the genus *Pseudomonas* were very enriched in bulb cloves (14%) and leaves (11.2%). *Salmonella* was preferentially identified in roots (8.8%) and bulb cloves (13%).

At the species level, *Streptomyces lividans* was the most dominant bacterial species in all six garlic tissues, ranging from 34.2% (inflorescences) to 71.1% (basal plates) (Figure 6D). The second most abundant bacterial species was *Escherichia coli*, which ranged from 4.2% (inflorescences) to 27.8% (roots). *Staphylococcus aureus* was preferentially identified from inflorescences (22.2%) and flowers (19.4%). *Pseudomonas tolaasii* was preferentially identified in leaves (11.1%), whereas *Staphylococcus cohnii* was preferentially identified from inflorescences (18.3%). *Salmonella enterica* was preferentially identified in roots (8.8%) and bulb cloves (13%), whereas *Pseudomonas aeruginosa* was preferentially identified from bulb cloves (12.4%).

### 2.7. Comparison of Identified Fungi in Different Garlic Tissues

**Figure 7:**
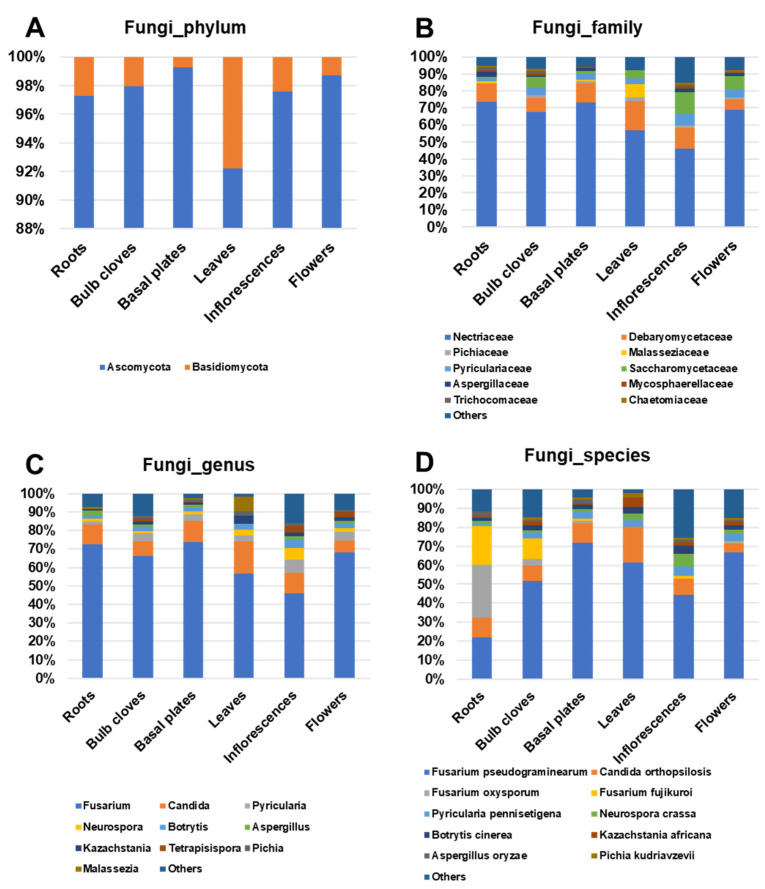
Proportion of identified fungal reads in each tissue at four different taxonomy levels, Proportion of identified bacterial reads in each tissue at four different taxonomy levels: (**A**) phylum, (**B**) family, (**C**) genus, and (**D**) species.

Ascomycota was the dominant fungus, ranging from 92.2% (leaves) to 99.3% (basal plates), whereas the proportion of Basidiomycota-associated reads ranged from 0.7% (basal plates) to 7.8% (leaves) (Figure 7A). The most abundant fungal families in six garlic tissues were Nectriaceae and Debaryomycetaceae (Figure 7B). Nectariaceae was the most dominant fungal family, ranging from 46.2% (inflorescences) to 73.5% (roots). Debaryomycetaceae was the second most dominant, ranging from 6.3% (flowers) to 17.3% (leaves). Fungi in the family Malasseziaceae were preferentially identified in leaves (7.8%) while Saccharomycetaceae were preferentially identified in inflorescences (12.7%), flowers (7.7%), and bulb cloves (6.5%). In addition, fungi in the family Pyriculariaceae were also frequently identified in most tissues, ranging from 1.8% (roots) to 6.9% (inflorescences).

Two fungal genera, Fusarium and Candida, were the most abundant fungal genera in the six garlic tissues (Figure 7C). Of the identified fungal genera, Fusarium was the most dominant fungal genus, ranging from 45.8% (inflorescences) to 73.6% (basal plates). Candida was the second most dominant fungus, ranging from 6.2% (flowers) to 17.3% (leaves). Six fungal genera, including Pyricularia, Neurospora, and Botrytis were highly present in inflorescences out of six tissues.

The most abundant fungal species was *Fusarium pseudograminearum*, which ranged from 22.1% (roots) to 71.7% (basal plates) (Figure 7D). In addition, two other *Fusarium* species, *F. oxysporum* and *F. fujikuroi*, were preferentially identified from roots (27.9% and 20.6%, respectively). *Candida orthopsilosis* was identified in all tissues ranging from 4.4% (flowers) to 18.8% (leaves). Three fungal species, *Pyricularia pennisetigena* (5%), *Neurospora crassa* (6.6%), and *Botrytis cinerea* (4.2%), were preferentially identified from inflorescences. *Kazachstania Africana* was abundantly identified in leaves (4.9%) out of the six tissues.

### 2.8. Comparison of Identified Viruses in Different Garlic Tissues

**Figure 8:**
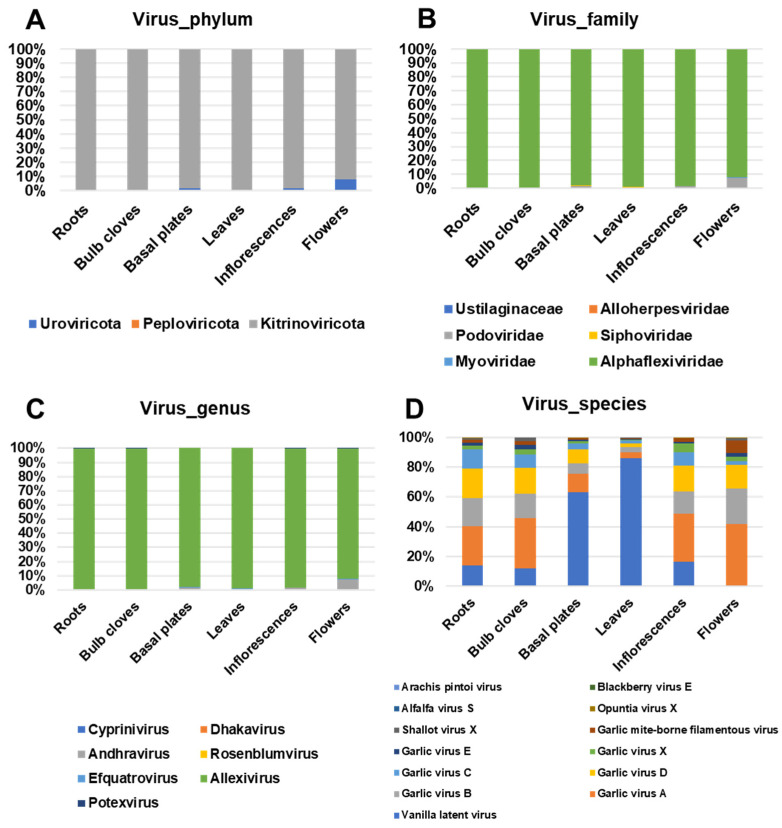
Proportion of identified viral reads in each tissue at four different taxonomy levels: (**A**) phylum, (**B**) family, (**C**) genus, and (**D**) species.

Most viral reads were identified as Kitrinoviricota, which ranged from 92% (flowers) to 99.7% (roots) (Figure 8A). In addition, we identified a small portion of viral reads associated with Uroviricota and Peploviricota. We identified viruses belonging to six viral families: Ustilaginaceae, Alloherpesviridae, Podoviridae, Siphoviridae, Myoviridae, and Alphaflexiviridae (Figure 8B). Of them, viruses in the family Alphaflexiviridae were the most abundant, ranging from 98.1% (basal plates) to 99.6% (roots). Viruses in the family Podoviridae were frequently identified in most tissues and in particular, the proportion of viral reads associated with Podoviridae was very high in flowers (7.4%). Six viral genera—Cyprinivirus, Dhakavirus, Andhravirus, Rosenblumvirus, Efquatrovirus, Allexivirus, and Potexvirus—were identified (Figure 8C). Of them, Allexivirus was the most dominant viral genus, ranging from 91.9% (flowers) to 99.4% (roots). Viruses belonging to the genus Andhravirus were also identified in all six tissues, ranging from 0.3% (roots) to 7.3% (flowers).

A total of 13 viral species were identified by Kraken2 (Figure 8D). The most abundant viral species was vanilla latent virus, which ranged from 0.1% (flowers) to 86.1% (leaves). However, the reads associated with vanilla latent virus were identified as sequences of other organisms, such as bacteria and animals. The next common viral species were allexiviruses, GarVA, GarVB, GarVC, GarVD, GarVE, GarVX, and garlic mite-borne filamentous virus. Of them, four viruses—GarA, GarVB, GarVC, and GarVD—were most abundantly present in all tissues. In addition, a recent study suggested that garlic mite-borne filamentous virus is conspecific with GarVA [19]. ShVX was preferentially identified in bulb cloves (2.1%) and flowers (1.6%). Although four viruses—opuntia virus X, alfalfa virus S, blackberry virus E, and arachis pintoi virus—were identified by Kraken2, we found that the reads associated with them were wrongly assigned.

### 2.9. Viromes in Six Different Garlic Tissues

**Figure 9:**
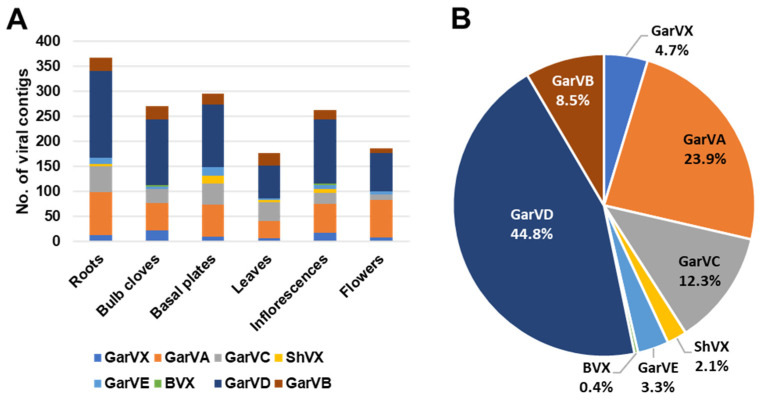
Identification of virus-associated contigs from six different garlic transcriptomes. (**A**) The number of viral contigs for each virus. (**B**) The proportion of identified viruses based on the number of viral contigs.

We found that Kraken2 did not properly reveal the garlic virome. Therefore, we analyzed the viromes in six different garlic tissues in detail by de novo assembly and BLASTX search. We de novo assembled each garlic transcriptome with the Trinity assembler. After BLASTX search against viral database, a total of eight viruses were identified. They were GarVA, GarVB, GarVC, GarVD, GarVE, GarvX, ShVX, and Botrytis virus X (BVX) (Table 3). We identified a total of 1559 viral contigs. Out of six tissues, we identified the highest number of viral contigs from roots (367 contigs), followed by basal plates (295 contigs), bulb cloves (271 contigs), and inflorescences (263 contigs) (Figure 9A). Based on the number of viral contigs, the most abundant virus was GarVD (44.8%), followed by GarVA (23.9%), GarVC (12.3%), GarVB (8.5), and GarVX (4.7%) (Figure 9B).

**Figure 10:**
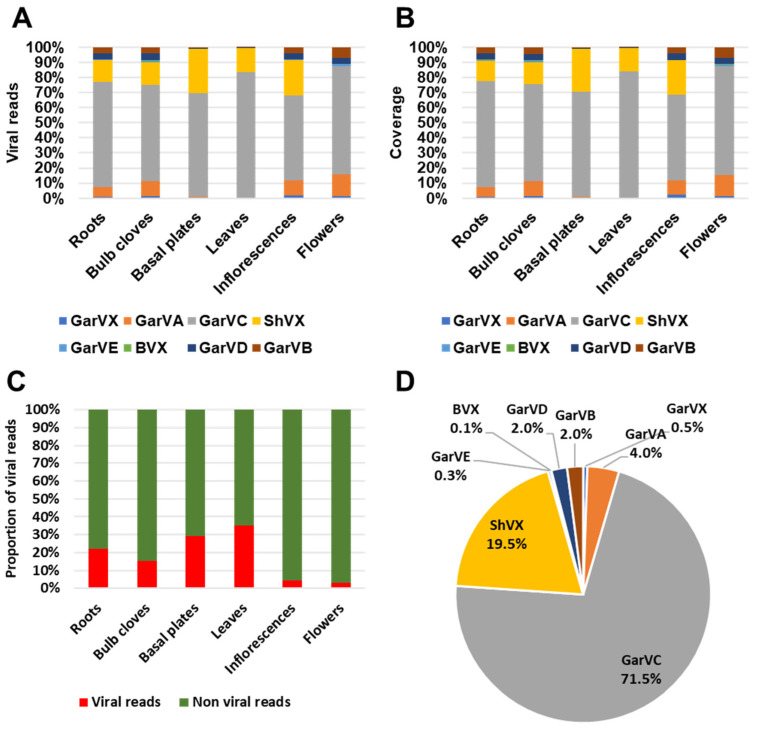
Proportion of identified viruses in six different garlic tissues. (**A**) Proportion of identified viruses based on viral reads. (**B**) Virus coverage in six different tissues. (**C**) Proportion of viral reads and non-viral reads in six different tissues. (**D**) Proportion of identified viruses based on viral reads obtained from all six tissues.

Next, we examined the number of viral reads and coverages for identified viruses (Figure 10). Based on viral reads, the most abundant virus was GarVC, which ranged from 56.1% (inflorescences) to 83.2% (leaves) (Figure 10A). The second most abundant virus was ShVX, which ranged from 0.3% (flowers) to 29.6% (basal plates). GarVA was preferentially enriched in roots (6.5%), bulb cloves (10.1%), inflorescences (9.8%), and flowers (14.3%), while the proportion of GarVA in basal plates and leaves was very low (0.7% and 0.2%, respectively). Similarly, GarVD was also highly enriched in four tissues: roots, bulb cloves, inflorescences, and flowers. BVX, a kind of mycovirus infecting fungus, was preferentially identified from roots (0.1%) and bulb cloves (0.4%).

Based on the viral coverages, GarVC was again the dominant virus, ranging from 58.2% (inflorescences) to 84.1% (leaves). ShVX was highly enriched in all tissues (13.5% to 28.7%) except flowers (0.3%) (Figure 10B). The genome sizes of identified viruses were similar; therefore, there was a significant difference of viral proportion between viral reads and coverages.

We analyzed the proportion of viral reads in each transcriptome (Figure 10C). The proportion of viral reads in leaves (35.3%) was the highest, followed by basal plates (29%), roots (21.9%), and bulb cloves (15.1%). Inflorescences and flowers contained relatively low number viral reads (4.5% and 3%, respectively). By combing all six virus-associated reads, GarVC (71.5%) was the most dominant virus, followed by ShVX (19.5%), GarVA (4%), GarVB (2%), and GarVD (2%) (Figure 10D).

We obtained a total of 14 viral genome sequences for four viruses: GarVA, GarVB, GarVC, and GarVX (Table 4). The two isolates of GarVA were derived from basal cloves and green leaves. BLASTN results showed that two GarVA isolates, BC and GL, showed sequence similarity to known GarVA isolate G122-2 from China, with 45% coverage and 78% nucleotide identity. GarVA encodes six open reading frames (ORFs). Two proteins—replicase and nucleic acid binding protein of GarVA isolates GL and BC—showed sequence similarity to GarVA while another four proteins—TGB1, TGB2, serine-rich protein, and coat protein—showed sequence similarity to GarVE. Four isolates for GarVB in this study derived from bulb cloves (three isolates) and inflorescence (one isolate) showed sequence similarity to the known isolate G119, with 100% coverage and 97% nucleotide identity (Table 4). All four GarVC isolates derived from green leaves were closely related with known isolate SW3.3A, with 99% coverage and 86% nucleotide identity. Four different GarVX isolates obtained from bulb cloves (one isolate), basal plates (one isolate), and flowers (two isolates) displayed sequence similarity to the known isolate G73-2, with 85% coverage and 77% nucleotide identity.

**Figure 11:**
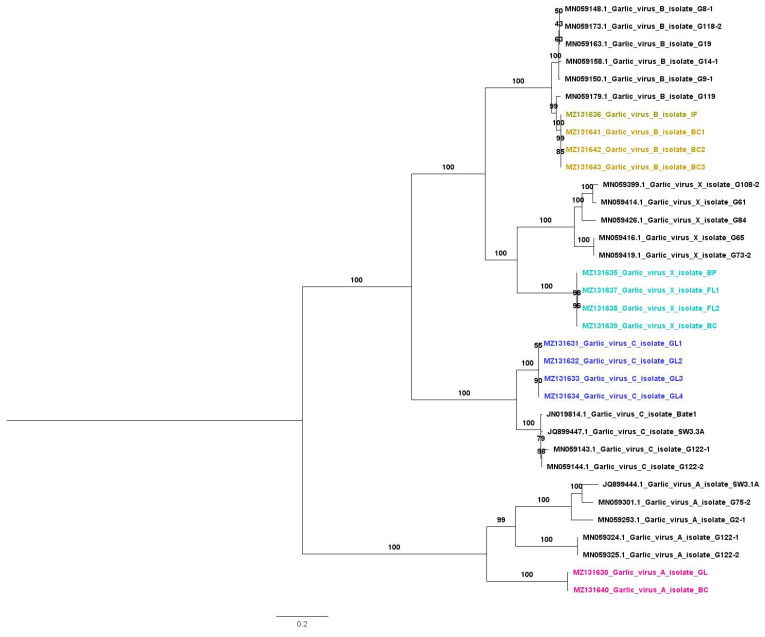
Phylogenetic relationships of 14 assembled garlic viral genomes. The 14 viral genomes sequences for GarVA, GarVB, GarVC, and GarVX were aligned with MAFFT using L-INS-i method. The phylogenetic tree was constructed by the maximum likelihood method and the GTR+F+I+G4 substitution model implemented in IQ-TREE. The phylogenetic tree is midpoint rooted using FigTree. Assembled viral genomes were indicated by different colors according to corresponding virus species.

Next, we generated a phylogenetic tree containing the 14 viral genomes in this study and 20 closely related viral genomes (Figure 11). Each allexivirus species was grouped together. Most of the four garlic virus species were grouped together with known garlic isolates from China.

## 3. Discussion

To date, marker genes such as rRNA and ITS for bacteria and fungi, respectively have been favorably used for microbiome study. In the case of viruses, which do not have a common marker sequence, DNA shot-gun sequencing or RNA-sequencing have been recently applied in a wide range of microbiome study. In this study, we demonstrated the usefulness of RNA-sequencing data and mRNA data derived from six different garlic tissues for microbiome study.

According to Kraken2 analysis, 57% of microbial reads were assigned to bacteria and 41% were assigned to viruses. Fungi only made up 1% of microbial reads. This result indicates that garlic transcriptomes contained a large number of reads from bacteria and viruses. As we expected, the microbial community was varied depending on the tissue types, although there was a dominant microorganism in each tissue. For example, *Actinobacteria* and *Proteobacteria* were dominant in most tissues except inflorescences and flowers, in which *Firmicutes* was preferentially enriched. In the case of fungi, *Ascomycota* was the most dominant fungal phylum in most tissues except leaves, in which *Basidiomycota* was the dominant fungal phylum. Similarly, the abundance of RNA viruses varied in different tissues. For example, viral RNAs mostly accumulated in leaves (35.3%) and basal plates (29%) and were less present in inflorescences (4.5%) and flowers (3%).

We found several pathogenic bacteria, such as *Staphylococcus*, *Escherichia*, *Salmonella*, and *Pseudomonas* species. Of the identified bacteria, *Streptomyces lividans* was the most dominant bacterial species in all six garlic tissues. Members of the genus *Streptomyces* frequently occur in the soil and play important roles in bioconversions [20,21]. *Staphylococcus aureus* is a bacterium commonly identified in the soil and causes staphylococcal food poisoning [22]. *Pseudomonas tolaasii* is a kind of Gram-negative soil bacteria that produces a toxin known as tolaasin, which causes brown blotch disease of *Agaricus bisporus* (Mushroom) [23]. *Pseudomonas aeruginosa* is frequently detected in many soil and raw vegetable samples and causes disease in plants, animals, and humans [24,25]. Most identified pathogenic bacteria in this study were identified not only in rhizosphere but also in phyllosphere regions. For example, a previous study demonstrated that *Salmonella enterica* transfers from soil to the phyllosphere of tomato fruits, thereby posing a public health threat when the *Salmonella enterica*–contaminated tomatoes are consumed [26]. *Escherichia coli* and *Salmonella enterica* were frequently identified from root and leaf vegetables grown in soils with incorporated bovine manure [27].

Several *Fusarium* species, such as *Fusarium pseudograminearum*, were identified as major fungal genera in all garlic tissues. It is already known that *Fusarium proliferatum* produces fumonisins, which cause dry rot disease and are regarded as important fungal pathogens of garlic [28]. *Fusarium proliferatum* causing rot of garlic bulbs has been reported in many countries such as Spain, North America, Serbia, and Germany [6,28,29,30]. However, we identified *F. pseudograminearum*, *F. oxysporum*, and *F. fujikuroi* species instead of *F. proliferatum*. We suppose that the database used for Kraken2 might be lacking the genome of *F. proliferatum*, which was recently published [31]. In addition, we identified several fungal pathogens. For example, *Pyricularia pennisetigena* causes leaf blast disease in plants of the *Poaceae* family [32]. *Botrytis cinerea* is known to cause grey mold disease for more than 200 crops in the world [33]. *Neurospora crassa* has also been identified in the carbohydrate-rich foodstuffs and residues of sugar-cane processing from the tropical and subtropical regions [34].

As previously reported, the garlic viromes were composed of different viral species in complex mixtures. In our study, most identified garlic viruses were allexiviruses. The members in the genus *Allexivirus* were composed of a single-stranded RNA genome with a poly(A) tail at the 3’ region. The genomes of allexiviruses are about 9 kb in length and encode six open reading frames (ORFs). Due to allexiviruses’ possession of a poly(A) tail, the obtained viral RNA from each transcriptome was very high. In addition, we could assemble 14 viral genomes for four viruses from the mRNA transcriptome that was obtained from a poly(A) selection for the library preparation. However, we failed to obtain a complete viral genome for ShVX, which was the second most dominant virus. It seems that a high abundance of viral reads does not guarantee the assembly of the target viral genomes. As shown in the phylogenetic tree, all assembled viral genomes in this study were grouped together with a small number of nucleotide differences, since they originated from the same plant via clonal propagation. Allexiviruses are known to be transmitted mechanically or by dry bulb mite (*Aceria tulipae*) [35]. Based on viral genome data and phylogenetic analysis, all identified allexiviruses were transmitted clonally.

GarVC was the most abundant viral species in all six tissues, although other members in the genus *Allexivirus*, such as GarVA, GarVB, and GarVD were also identified. However, the abundance of viral RNAs for allexiviruses excluding GarVC was relatively low in basal plates and leaves. This result suggests that GarVC might be very competitive compared to other allexiviruses in basal plates and leaves that have a high replication ability. Although GarVC was the dominant virus in our study, it is not clear whether GarVC could also be dominant in other garlic samples. Further study should be performed using diverse garlic cultivars from different geographical regions. ShVX was the second most abundant viral species in most tissues except flowers, which had a low abundance of ShVX. Thus, it is important that the proper selection of plant tissue be required for microbial diagnostics and microbiome study.

It is very hard to extract only viral nucleic acids from virus-infected plant samples. Several methods depending on the target viruses have been used to enrich viral nucleic acids. Surprisingly, we found that the proportion of viral reads in the six garlic tissues was very high compared to those of other studies. This result suggests that the abundance of the RNA viruses infecting garlic was very high, which indicates a high replication of those RNA viruses.

To date, there are several tools for taxonomy classification of microbiomes with shotgun metagenomics data [36,37]. Of the known tools, we selected Kraken2, which uses a k-mer counting approach for elucidation of the microbial populations from RNA-sequencing data. As previously described, Kraken2 generated accurate taxonomic identification for bacteria with very fast speed [37,38]. However, only a few studies have used Kraken2 for fungal and viral microbiome studies [39,40]. In this study, we have shown that Kraken2 is a powerful tool for identifying bacteria, fungi, and viruses. In particular, Kraken2 revealed very reliable taxonomy classification results for bacteria; however, there were some limitations in the identification of fungi and viruses. The first problem was that there were lots of unassigned reads by Kraken2 analysis. In the case of viruses, Kraken2 identified 395,096 viral reads while BWA-based mapping resulted in identification of 4,923,406 viral reads. The second problem was that Kraken2 wrongly assigned several viruses with a low number of reads at the viral species level. For example, arachis pintoi virus, blackberry virus E, alfalfa virus S, and opuntia virus X have not been reported in garlic, and garlic mite-borne filamentous virus should be conspecific with GarVA based on recent study [19]. The big problem was with vanilla latent virus, which is phylogenetically close to allexiviruses. Vanilla latent virus was identified with a high number of reads by Kraken2 in this study. However, BLASTN results of reads associated with vanilla latent virus showed that they were not vanilla latent virus but partial reads from bacteria or insects. In fact, it might be very difficult for people who do not have good knowledge of plant viruses and bioinformatic data analysis to find false positive results. Based on these results, we suggest that additional experiments be performed to eliminate false positive results.

In this study, we revealed tissue-specific microbial communities for bacteria, fungi, and viruses using RNA-sequencing data via Kraken2. We found that Kraken2 was a very powerful and efficient tool for microbiome study using RNA-sequencing data, although there are still some limitations for virome study.

## 4. Materials and Methods

### 4.1. Plant Materials, Library Preparation, and RNA-Sequencing

The data in this study were derived from a previous garlic transcriptome study [17]. Only a few garlic genotypes can produce flowers, so we selected garlic transcriptome data of fertile garlic containing flower tissues. The fertile garlic cultivar No. 87 was derived from a single seed. The freshly harvested garlic bulbs were stored. The healthy cloves were planted. Six different tissues—cloves, basal plates, green leaves, roots, inflorescences, and flowers—with three biological replicates were collected from March to July 2013 at the ARO, The Volcani Center, Bet Dagan, Israel.

The extracted total RNAs were used for library preparation using Illumina’s TruSeq RNA Sample Prep kit with random primers (Illumina, San Diego, CA, USA). The libraries were paired-end (250 bp × 2) sequenced using the MiSeq platform.

### 4.2. Trimming Poor-Quality Reads and Reads from the Garlic Plants

We obtained raw sequences from the SRA database with the following accession numbers: SRR1219646, SRR1220207, SRR1219644, SRR1219535, SRR1219796, and SRR1219989. We performed quality control of raw data to eliminate low-quality bases (Phred quality score < 20) and reads less than 50 bp using the BBDuk ver. 37.33 program (https://jgi.doe.gov/data-and-tools/bbtools/bb-tools-user-guide/bbduk-guide/) (accessed on 23 January 2021). We generated a database containing garlic cDNAs, chloroplast (NC_031829.1), and the mitochondrial genome of Allium cepa (NC_030100.1) [41]. The data in this study were derived from RNAs, so we used cDNA instead of genomic DNA. In addition, the mitochondrial genome of garlic was not currently available, so we used the mitochondrial genome of onion (Allium cepa). Next, we deleted garlic-associated reads by mapping raw sequence reads on the generated garlic database using BBDuk ver. 37.33. Finally, after filtering poor-quality reads and reads from garlic, we used clean reads for microbiome study.

### 4.3. Microbiome Analysis by Kraken2 and Bracken

For microbiome study, we used the Kraken2 program [18]. The clean paired-end sequence reads stored in FASTQ format were used for Kraken2 analysis against the PlusPFP database containing archaea, bacteria, viral, plasmid, human, UniVec_core, protozoa, fungi, and plants (https://benlangmead.github.io/aws-indexes/k2) (accessed on 23 January 2021). To calculate the abundance of identified species from the Kraken2 analysis, we used the Bracken program [42]. Results of the Kraken2/Bracken were analyzed using the Pavian program (https://fbreitwieser.shinyapps.io/pavian/) (accessed on 23 January 2021) [43].

### 4.4. Garlic Virome Analysis

The filtered clean reads in each library were used for de novo transcriptome assembly using Trinity ver. 2.7.8a with default parameters [44]. After that, we conducted BLASTX (E-value less than 0.001) against the viral genome database. The obtained virus-associated contigs were again subjected to BLASTN search (E-value less than 0.00001) against NCBI’s nucleotide database. Based on BLASTN results, we removed non-viral contigs.

Based on de novo assembly and BLAST search, we obtained several complete or nearly complete viral genomes covering all open reading frames (ORFs). Based on the BLAST results, the virus-associated contigs in each library were subjected to ORF prediction using the ORF Finder program (https://www.ncbi.nlm.nih.gov/orffinder/) (accessed on 23 January 2021). To calculate the abundance of identified viruses, raw sequence reads were mapped on the identified virus genome using the Burrows–Wheeler Aligner (BWA) program with default parameters [45]. Fourteen complete viral genome sequences covering whole ORFs were deposited in NCBI’s GenBank database with respective accession numbers.

### 4.5. Phylogenetic Analyses

The obtained 14 viral genome sequences in this study were subjected to BLASTN search to identify known viral genome sequences showing sequence similarity. For each viral species, we selected five virus isolates. All assembled viruses were members of the genus Allexivirus. Therefore, all complete viral genomes in this study and homologous known viral isolates were used for phylogenetic analysis. All complete viral genome nucleotide sequences were aligned by MAFFT ver. 7.310 (17 March 2017) with the G-INS-i (accurate) strategy [46]. The aligned sequences were trimmed by the trimAL program with the automated method (http://trimal.cgenomics.org/) (accessed on 23 January 2021) [47]. We selected the best-fitting substitution model using IQ-TREE [48]. We generated the phylogenetic tree using the IQ-TREE program with the maximum likelihood method, selected substitution model, and ultrafast bootstrap according to the manufacturers’ instructions. The generated phylogenetic tree was visualized using the phylogenetic tree (http://tree.bio.ed.ac.uk/software/figtree/) (accessed on 23 January 2021).

## Figures and Tables

**Table 1:** Summary of reads after removing poor-quality reads and garlic-associated reads. Total reads were subjected to trimming to remove poor-quality reads (Filtered reads) and garlic-associated reads (nuclear, chloroplast, and mitochondrial sequences). After trimming, clean reads were subjected to microbiome analyses.

Accession Number	SRR1219646	SRR1220207	SRR1219644	SRR1219535	SRR1219796	SRR1219989
Tissue	Roots	Bulb cloves	Basal plates	Leaves	Inflorescences	Flowers
Total reads	9,475,970	9,555,462	10,015,640	7,459,884	12,027,808	8,325,776
Garlic reads	5,619,202 (59.30%)	6,375,736 (66.72%)	5,807,458 (57.98%)	3,955,182 (53.02%)	8,826,442 (73.38%)	6,189,796 (74.34%)
Filtered reads	819,162 (8.64%)	733,549 (7.68%)	897,537 (8.96%)	742,660 (9.96%)	630,865 (5.25%)	393,386 (4.72%)
Removed reads	5,619,482 (59.30%)	6,376,048 (66.73%)	5,807,728 (57.99%)	3,955,384 (53.02%)	8,826,770 (73.39%)	6,190,034 (74.35%)
Clean reads	3,856,488 (40.70%)	3,179,414 (33.27%)	4,207,912 (42.01%)	3,504,500 (46.98%)	3,201,038 (26.61%)	2,135,742 (25.65%)

**Table 2:** Alpha diversity of identified microbiomes at species level in six different garlic tissues. Three different diversity indexes (Shannon, Simpson, and inverse Simpson) were used.

Tissue	Shannon	Simpson	Inverse Simpson
Roots	2.4339584	0.880831201	8.391458226
Bulb cloves	2.6770221	0.892296316	9.284733497
Basal plates	1.8485327	0.696672906	3.29677111
Leaves	1.8102818	0.739783711	3.842956962
Inflorescences	2.9469045	0.901497864	10.15206406
Flowers	2.2674492	0.789839261	4.758262668

**Table 3:** Summary of identified viruses from garlic transcriptomes. The number indicates the number of virus-associated contigs in each garlic tissue.

Virus	Abbreviation	Accession No.	Viral Genome Size	Roots	Bulb Cloves	Basal Plates	Leaves	Inflorescences	Flowers
Garlic virus X	GarVX	NC_001800.1	8106	13	21	9	6	17	7
Garlic virus A	GarVA	NC_003375.1	8660	86	56	64	34	58	75
Garlic virus C	GarVC	NC_003376.1	8405	51	27	43	38	21	12
Shallot virus X	ShVX	NC_003795.1	8832	5	1	15	4	8	0
Garlic virus E	GarVE	NC_004012.1	8451	12	4	18	3	9	6
Botrytis virus X	BVX	NC_005132.1	6966	0	3	0	0	3	0
Garlic virus D	GarVD	NC_022961.1	8424	173	131	124	67	127	76
Garlic virus B	GarVB	NC_025789.1	8336	27	28	22	25	20	10

**Table 4:** BLASTN results of 14 assembled viral genomes. The 14 assembled viral genome sequences were subjected to BLASTN search against viral database. Acc. No. indicates accession number of assembled viral genomes in GenBank.

Name of Virus	Acc. No.	Matched Viral Genome	Acc. No.	Coverage	Identity
Garlic virus A isolate BC	MZ131640	Garlic virus A isolate G122-2	MN059325.1	45%	78%
Garlic virus A isolate GL	MZ131630	Garlic virus A isolate G122-2	MN059325.1	45%	78%
Garlic virus B isolate BC1	MZ131641	Garlic virus B isolate G119	MN059179.1	100%	97%
Garlic virus B isolate BC2	MZ131642	Garlic virus B isolate G119	MN059179.1	100%	97%
Garlic virus B isolate BC3	MZ131643	Garlic virus B isolate G119	MN059179.1	100%	97%
Garlic virus B isolate IF	MZ131636	Garlic virus B isolate G119	MN059179.1	100%	97%
Garlic virus C isolate GL1	MZ131631	Garlic virus C isolate SW3.3A	JQ899447.1	99%	86%
Garlic virus C isolate GL2	MZ131632	Garlic virus C isolate SW3.3A	JQ899447.1	99%	86%
Garlic virus C isolate GL3	MZ131633	Garlic virus C isolate SW3.3A	JQ899447.1	99%	86%
Garlic virus C isolate GL4	MZ131634	Garlic virus C isolate SW3.3A	JQ899447.1	99%	86%
Garlic virus X isolate BC	MZ131639	Garlic virus X isolate G73-2	MN059419.1	85%	77%
Garlic virus X isolate BP	MZ131635	Garlic virus X isolate G73-2	MN059419.1	84%	77%
Garlic virus X isolate FL1	MZ131637	Garlic virus X isolate G73-2	MN059419.1	85%	77%
Garlic virus X isolate FL2	MZ131638	Garlic virus X isolate G73-2	MN059419.1	85%	77%

## Data Availability

The 14 viral genome sequences obtained from this study were deposited in GenBank, NCBI, with respective accession numbers.

## References

[1] Stoyanova M., Moncheva P., Bogatzevska N. (2012). Occurrence of phytopathogenic bacteria of Enterobacteriaceae family in bulbs of cultural and ornamental plants. Sci. Technol..

[2] Sawada H., Horita H., Nishimura F., Mori M. (2020). *Pseudomonas salomonii*, another causal agent of garlic spring rot in Japan. J. Gen. Plant Pathol..

[3] Roumagnac P., Gagnevin L., Gardan L., Sutra L., Manceau C., Dickstein E., Jones J.B., Rott P., Pruvost O. (2004). Polyphasic characterization of xanthomonads isolated from onion, garlic and Welsh onion (*Allium* spp.) and their relatedness to different *Xanthomonas* species. Int. J. Syst. Evol. Microbiol..

[4] Ulacio-Osorio D., Zavaleta-Mejía E., Martínez-Garza A., Pedroza-Sandoval A. (2006). Strategies for management of *Sclerotium cepivorum* Berk. in garlic. J. Plant Pathol..

[5] Onaebi C., UGWUJA F., Okoro A., Amujiri A., Ivoke M. (2020). Mycoflora associated with post-harvest rot of onion (*Allium cepa*) and garlic (*Allium sativum*) bulbs. Res. Crop..

[6] Stankovic S., Levic J., Petrovic T., Logrieco A., Moretti A. (2007). Pathogenicity and mycotoxin production by *Fusarium proliferatum* isolated from onion and garlic in Serbia. Eur. J. Plant Pathol..

[7] Zhang J., Li G., Jiang D. (2009). First report of garlic leaf blight caused by *Botrytis porri* in China. Plant Dis..

[8] Lee H.B., Kim C.-J., Yu S.H. (2002). First report of bulb canker of garlic caused by *Embellisia allii* in Korea. Mycobiology.

[9] Worku Y., Dejene M. (2012). Effects of garlic rust (*Puccinia allii*) on yield and yield components of garlic in Bale highlands, south eastern Ethiopia. J. Plant Pathol. Microbiol..

[10] Fajardo T.V., Nishijima M., Buso J.A., Torres A.C., Ávila A.C., Resende R.O. (2001). Garlic viral complex: Identification of potyviruses and carlavirus in central Brazil. Fitopatol. Bras..

[11] Parrano L., Afunian M., Pagliaccia D., Douhan G., Vidalakis G. (2012). Characterization of viruses associated with garlic plants propagated from different reproductive tissues from Italy and other geographic regions. Phytopathol. Mediterr..

[12] Walters W., Hyde E.R., Berg-Lyons D., Ackermann G., Humphrey G., Parada A., Gilbert J.A., Jansson J.K., Caporaso J.G., Fuhrman J.A. (2016). Improved bacterial 16S rRNA gene (V4 and V4-5) and fungal internal transcribed spacer marker gene primers for microbial community surveys. Msystems.

[13] Ellis R.J., Morgan P., Weightman A.J., Fry J.C. (2003). Cultivation-dependent and-independent approaches for determining bacterial diversity in heavy-metal-contaminated soil. Appl. Environ. Microbiol..

[14] Knief C. (2014). Analysis of plant microbe interactions in the era of next generation sequencing technologies. Front. Plant Sci..

[15] Garmaeva S., Sinha T., Kurilshikov A., Fu J., Wijmenga C., Zhernakova A. (2019). Studying the gut virome in the metagenomic era: Challenges and perspectives. BMC Biol..

[16] Gaafar Y.Z.A., Ziebell H. (2020). Comparative study on three viral enrichment approaches based on RNA extraction for plant virus/viroid detection using high-throughput sequencing. PLoS ONE.

[17] Kamenetsky R., Faigenboim A., Mayer E.S., Michael T.B., Gershberg C., Kimhi S., Esquira I., Shalom S.R., Eshel D., Rabinowitch H.D. (2015). Integrated transcriptome catalogue and organ-specific profiling of gene expression in fertile garlic (*Allium sativum* L.). BMC Genom..

[18] Wood D.E., Lu J., Langmead B. (2019). Improved metagenomic analysis with Kraken 2. Genome Biol..

[19] Geering A.D., McTaggart A.R. (2019). Questions surrounding the taxonomic validity of the species Garlic mite-borne filamentous virus (genus Allexivirus). Arch. Virol..

[20] Gao Q., Piret J., Adrio J., Demain A. (2003). Performance of a recombinant strain of *Streptomyces lividans* for bioconversion of penicillin G to deacetoxycephalosporin G. J. Ind. Microbiol. Biotechnol..

[21] Park J.-W., Lee J.-K., Kwon T.-J., Yi D.-H., Kim Y.-J., Moon S.-H., Suh H.-H., Kang S.-M., Park Y.-I. (2003). Bioconversion of compactin into pravastatin by *Streptomyces* sp.. Biotechnol. Lett..

[22] Johler S., Tichaczek-Dischinger P.S., Rau J., Sihto H.-M., Lehner A., Adam M., Stephan R. (2013). Outbreak of Staphylococcal food poisoning due to SEA-producing *Staphylococcus aureus*. Foodborne Pathog. Dis..

[23] Soler-Rivas C., Jolivet S., Arpin N., Olivier J., Wichers H. (1999). Biochemical and physiological aspects of brown blotch disease of *Agaricus bisporus*. FEMS Microbiol. Rev..

[24] Green S.K., Schroth M.N., Cho J.J., Kominos S.D., Vitanza-Jack V.B. (1974). Agricultural plants and soil as a reservoir for *Pseudomonas aeruginosa*. Appl. Microbiol..

[25] Driscoll J.A., Brody S.L., Kollef M.H. (2007). The epidemiology, pathogenesis and treatment of *Pseudomonas aeruginosa* infections. Drugs.

[26] Barak J.D., Liang A.S. (2008). Role of soil, crop debris, and a plant pathogen in *Salmonella enterica* contamination of tomato plants. PLoS ONE.

[27] Natvig E.E., Ingham S.C., Ingham B.H., Cooperband L.R., Roper T.R. (2002). *Salmonella enterica* serovar Typhimurium and *Escherichia coli* contamination of root and leaf vegetables grown in soils with incorporated bovine manure. Appl. Environ. Microbiol..

[28] Palmero D., De Cara M., Iglesias C., Moreno M., Gonzalez N., Tello J. (2010). First report of *Fusarium proliferatum* causing rot of garlic bulbs in Spain. Plant Dis..

[29] Dugan F., Hellier B., Lupien S. (2003). First report of *Fusarium proliferatum* causing rot of garlic bulbs in North America. Plant Pathol..

[30] Seefelder W., Gossmann M., Humpf H.-U. (2002). Analysis of fumonisin B1 in Fusarium proliferatum-infected asparagus spears and garlic bulbs from Germany by liquid chromatography− electrospray ionization mass spectrometry. J. Agric. Food Chem..

[31] Almiman B.F., Shittu T.A., Muthumeenakshi S., Baroncelli R., Sreenivasaprasad S. (2018). Genome sequence of the mycotoxigenic crop pathogen *Fusarium proliferatum* strain ITEM 2341 from date palm. Microbiol. Resour. Announc..

[32] Reges J.T.d.A., Negrisoli M.M., Dorigan A.F., Castroagudín V.L., Maciel J.L.N., Ceresini P.C. (2016). *Pyricularia pennisetigena* and *P. zingibericola* from invasive grasses infect signal grass, barley and wheat. Pesqui. Agropecu. Trop..

[33] Williamson B., Tudzynski B., Tudzynski P., Van Kan J.A. (2007). *Botrytis cinerea*: The cause of grey mould disease. Mol. Plant Pathol..

[34] Kuo H.-C., Hui S., Choi J., Asiegbu F.O., Valkonen J.P., Lee Y.-H. (2014). Secret lifestyles of *Neurospora crassa*. Sci. Rep..

[35] Sarwar M. (2020). Mite (*Acari Acarina*) vectors involved in transmission of plant viruses. Applied Plant Virology.

[36] Tamames J., Cobo-Simón M., Puente-Sánchez F. (2019). Assessing the performance of different approaches for functional and taxonomic annotation of metagenomes. BMC Genom..

[37] Simon H.Y., Siddle K.J., Park D.J., Sabeti P.C. (2019). Benchmarking metagenomics tools for taxonomic classification. Cell.

[38] Kibegwa F.M., Bett R.C., Gachuiri C.K., Stomeo F., Mujibi F.D. (2020). A comparison of two DNA metagenomic bioinformatic pipelines while evaluating the microbial diversity in feces of Tanzanian small holder dairy cattle. BioMed Res. Int..

[39] Maza-Márquez P., Lee M.D., Bebout B.M. (2021). The abundance and diversity of fungi in a hypersaline microbial mat from Guerrero Negro, Baja California, México. J. Fungi.

[40] Abouelkhair M.A. (2020). Non-SARS-CoV-2 genome sequences identified in clinical samples from COVID-19 infected patients: Evidence for co-infections. PeerJ.

[41] Sun X., Zhu S., Li N., Cheng Y., Zhao J., Qiao X., Lu L., Liu S., Wang Y., Liu C. (2020). A chromosome-Level genome assembly of garlic (*Allium sativum*) provides insights into genome evolution and allicin biosynthesis. Mol. Plant.

[42] Lu J., Breitwieser F.P., Thielen P., Salzberg S.L. (2017). Bracken: Estimating species abundance in metagenomics data. PeerJ Comput. Sci..

[43] Breitwieser F.P., Salzberg S.L. (2020). Pavian: Interactive analysis of metagenomics data for microbiome studies and pathogen identification. Bioinformatics.

[44] Grabherr M.G., Haas B.J., Yassour M., Levin J.Z., Thompson D.A., Amit I., Adiconis X., Fan L., Raychowdhury R., Zeng Q. (2011). Trinity: Reconstructing a full-length transcriptome without a genome from RNA-Seq data. Nat. Biotech..

[45] Li H., Durbin R. (2010). Fast and accurate long-read alignment with Burrows–Wheeler transform. Bioinformatics.

[46] Katoh K., Standley D.M. (2013). MAFFT multiple sequence alignment software version 7: Improvements in performance and usability. Mol. Biol. Evol..

[47] Capella-Gutiérrez S., Silla-Martínez J.M., Gabaldón T. (2009). trimAl: A tool for automated alignment trimming in large-scale phylogenetic analyses. Bioinformatics.

[48] Minh B.Q., Schmidt H.A., Chernomor O., Schrempf D., Woodhams M.D., Von Haeseler A., Lanfear R. (2020). IQ-TREE 2: New models and efficient methods for phylogenetic inference in the genomic era. Mol. Biol. Evol..

